# ROS-Responsive Berberine Polymeric Micelles Effectively Suppressed the Inflammation of Rheumatoid Arthritis by Targeting Mitochondria

**DOI:** 10.1007/s40820-020-0410-x

**Published:** 2020-03-20

**Authors:** Xing-xing Fan, Meng-ze Xu, Elaine Lai-Han Leung, Cai Jun, Zhen Yuan, Liang Liu

**Affiliations:** 1 State Key Laboratory of Quality Research in Chinese Medicine, Macau Institute For Applied Research in Medicine and Health, Macau University of Science and Technology, Avenida Wai Long, Taipa, Macau SAR China; 2 Faculty of Health Sciences, University of Macau, Taipa, Macau SAR China

**Keywords:** Rheumatoid arthritis, Reactive oxygen species, Nanoparticles, Berberine, Oxygen consumption rate

## Abstract

Rheumatoid arthritis (RA) is an autoimmune disease, which attacks human joint system and causes lifelong inflammatory condition. To date, no cure is available for RA and even the ratio of achieving remission is very low. Hence, to enhance the efficacy of RA treatment, it is essential to develop novel approaches specifically targeting pathological tissues. In this study, we discovered that RA synovial fibroblasts exhibited higher reactive oxygen species (ROS) and mitochondrial superoxide level, which were adopted to develop ROS-responsive nano-medicines in inflammatory microenvironment for enhanced RA treatment. A selenocystamine-based polymer was synthesized as a ROS-responsive carrier nanoplatform, and berberine serves as a tool drug. By assembling, ROS-responsive berberine polymeric micelles were fabricated, which remarkably increased the uptake of berberine in RA fibroblast and improved in vitro and in vivo efficacy ten times higher. Mechanistically, the anti-RA effect of micelles was blocked by the co-treatment of AMPK inhibitor or palmitic acid, indicating that the mechanism of micelles was carried out through targeting mitochondrial, suppressing lipogenesis and finally inhibiting cellular proliferation. Taken together, our ROS-responsive nano-medicines represent an effective way of preferentially releasing prodrug at the inflammatory microenvironment and improving RA therapeutic efficacy.

**Electronic supplementary material:**

The online version of this article (10.1007/s40820-020-0410-x) contains supplementary material, which is available to authorized users.

## Article Highlights

- Reactive oxygen species (ROS)-responsive nano-medicines represent an effective way of preferentially releasing prodrug at the inflammatory microenvironment and improving rheumatoid arthritis therapeutic efficacy.
- The combination of ROS-responsive carrier nanoplatform and berberine is a potential agent for treating rheumatoid arthritis.

**Electronic supplementary material:**

The online version of this article (10.1007/s40820-020-0410-x) contains supplementary material, which is available to authorized users.

## Graphical abstract

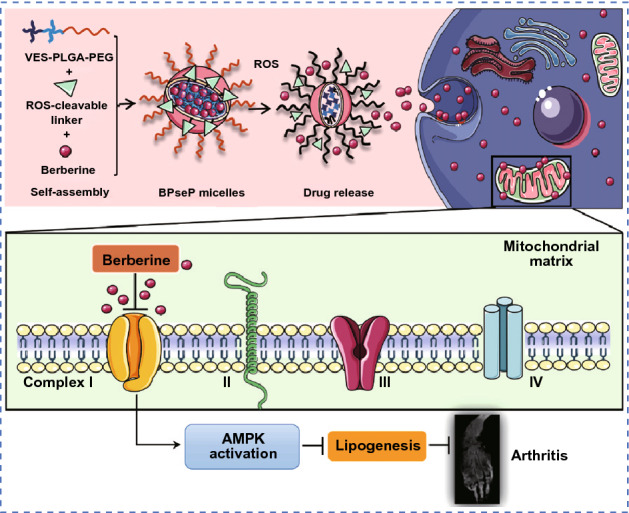

## Introduction

Rheumatoid arthritis (RA) is a chronic arthritis caused by the malfunction of immune system and is estimated to affect nearly 0.5–1% of the adults worldwide [1, 2]. As the disease progresses, it might cause symptoms such as inflammation, swelling and pain. Although a bunch of factors contribute to its development, including smoking, gender, age, obesity, and genetic traits, the hallmark of RA progressively damages the joint system [3].The downside of this disease is that no cure is presently available for RA. However, drugs with sufficiently high therapeutic index can help relieve symptoms such as pain and inflammation and significantly slow its progression.

Interestingly, present clinical medications for RA mainly involve four categories of drugs: nonsteroidal anti-inflammatory drugs (NSAIDs), glucocorticoids, nonbiologic disease-modifying antirheumatic drugs (DMARDs), and biologic DMARDs [2]. Although current treatments for RA have generated substantial success, 30% of RA patients who receive the drug intervention still show insufficient response to the first-line therapy. For example, regarding methotrexate (MTX) [4], 30% of patients exhibit intolerance of MTX during the 1-year treatment due to its odious side effects and systematic complication [5]. In anticipation of new drugs with the potential to improve the efficacy of therapy, it is essential to develop novel agents and approaches to preferentially aggregate and specifically target the affected tissues.

In addition, it is widely recognized that synovial cells of RA express genes and erosive enzymes that promote joint degradation [6]. Meanwhile, theranostic nano-agents have emerged as a promising nanotechnology for targeting delivery system to many diseases [7, 8], including RA synovial cells for enhanced RA treatment [9]. The conjugation of a targeting ligand to chemically modified nanoparticles will allow for direct selective binding to cell types. In particular, numerous targets (such as selectins, folate receptor, matrix metalloproteases, and Fc-γ receptor) have been successfully utilized to facilitate the specific delivery of drugs and improve the therapeutic efficacy [10–14]. In this study, a novel inflammation-triggerable strategy is exploited, which can induce constructed nano-medicines to preferentially accumulate in the inflammatory microenvironment for targeted RA therapy.

Further, due to the demands of high proliferation of RA inflamed synovium, metabolism abnormalities are considered as a common feature of RA, which might be developing into a potentially therapeutic tool for the intervention of RA [15]. To date, a number of metabolism-mediating compounds have been reported to successfully alleviate the diseased activity [16, 17]. More importantly, berberine, as an alkaloid isolated from plant *Coptis chinensis*, exhibits multiple pharmacological activities [18]. In particular, its metabolism-regulating function and associated mechanism have been explored by a number of experimental studies [19], in which its anti-RA effect was also carefully inspected [20–22]. Berberine, as an effective agent for suppressing the progress of RA by targeting mitochondrial oxidative phosphorylation, can serve as a unique tool drug for the treatment for RA [21].

More specifically, reactive oxygen species (ROS) are usually generated in the diseased tissues due to increased metabolic rate and peroxisome activities, elevated cell signaling, and dysfunction of mitochondria. Interestingly, we discovered that ROS was extremely up-regulated in RA samples, no matter with or without MTX resistance. Consequently, in this study, we design a novel multifunctional nanoplatform by assembling ROS-responsive delivery system with anti-RA agent berberine, which can produce an inflammation-targeted nano-medicine. The proof-of-concept application of targeted nano-medicine is inspected in a mouse RA model. Meanwhile, in vivo therapy was performed, in which we discover the developed inflammation-targeted nano-medicine showed high efficacy of RA treatment. The nanoplatform constructed here should thus be a new category of nano-medicine for enhanced RA treatment, which paves a new avenue for their potential clinical application.

## Materials and Methods

### Materials

Poly(lactic-co-glycolic acid) (PLGA), (LA/GA = 50:50, MW 5000) was purchased from Xi’an Ruixi Biological Technology, vitamin E succinate was purchased from TCI (Shanghai) Development Co., Ltd. and 2,2′-Diselanediyldiethanamine dihydrochloride was bought from Jiangsu Aikonchem Co. Ltd. Dicyclohexylcarbodiimide (DCC) and 4-dimethylaminopyridine (DMAP) were ordered from Sigma-Aldrich. 1-Ethyl-3-(3-dimethylaminopropyl) carbodiimide (EDC), *N*-hydroxy succinimide (NHS), compound C and berberine were purchased from Sigma-Aldrich. Hydrogen peroxide (H_2_O_2_) was ordered from VWR International, LLC. Methoxy polyethylene glycol–propionic acid (mPEG–COOH, MW 2000) and methoxy poly(ethylene glycol) amine (mPEG-NH2, MW 2000) were purchased from Biomatrix Inc. Palmitic acid was acquired from Cayman. All chemicals were used without further purification. Primary antibodies were purchased from cell signaling technology, and fluorescein-conjugated second antibodies were purchased from Odyssey.

### Cell Lines and Patients’ Serum Samples

HFLS, HFLS-OA and HFLS-RA were purchased from Cell Application, Inc. MTX-resistant HFLS-RA was acquired by gradually increasing the MTX concentration in culture medium up 500 μM. All cells were cultured with Human Synoviocyte Growth Medium and cultivated at 37 °C in a 5% CO_2_ incubator. Patients’ serum samples were collected from Guangdong Provincial Hospital of Chinese Medicine following the hospital guidelines, and patients signed informed consent in all cases.

### Synthesis and Characterization of VPseP Co-polymers

The vitamin E succinate–poly (lactic-co-glycolic acid)–selenocystamine dihydrochloride–methoxy poly(ethylene glycol) co-polymers (VES-PLGA-Se-Se-mPEG, VPseP) and methoxy poly(ethylene glycol)–poly (lactic-co-glycolic acid) co-polymers (VPP) were synthesized mainly via esterification and amide reaction. Briefly, 1 mmol vitamin E succinate (VES), 1.2 mmol DCC, 0.1 mmol DMAP, and 3 mmol TEA were dissolved in dichloromethane to activate the carboxyl group of VES followed by adding 1 mmol OH-PLGA-COOH drop by drop to generate the VES-PLGA-COOH, which subsequently reacted with 2,2′-diselanediyldiethanamine dihydrochloride to acquire VES-PLGA-Se-Se-NH_2_ and then mPEG-COOH to yield VES-PLGA-Se-Se-mPEG through EDC/NHS-mediated amide reaction, all of which were purified by co-precipitation with ether, dialysis, and lyophilization. The structures of VES–PLGA–Se–Se–PEG, mPEG–COOH, VES–PLGA–Se–Se–NH_2_, NH_2_–(CH_2_)_2_–Se–Se–(CH_2_)_2_–NH_2_, VES–PLGA–COOH, VES–COOH, OH–PLGA–COOH were all characterized by ^1^H–NMR (Bruker AV-400 instrument, Germany), FTIR (Perkin Elmer Precise Spectrum 100 Infrared Spectrometer, US). The vitamin E succinate–poly (lactic-co-glycolic acid)–methoxy poly(ethylene glycol) co-polymers were synthesized with the similar procedure except for using methoxy poly(ethylene glycol) amine (mPEG-NH_2_) as the reactant instead of mPEG-COOH.

### Preparation and Characterization of BPseP Micelles

The berberine-loaded PseP micelles (BPseP) were prepared by dialysis method. Briefly, uniformly mixure of 500 μL PseP (10 mg mL^−1^) in dimethyl sulfoxide with 200 μL berberine (5 mg mL^−1^) in dimethyl sulfoxide then was added dropwise into 8 mL phosphate buffer saline when fierce magnetic stirring. Afterward, the balanced mixture was dialyzed against ultrapure water using a sealed dialysis bag (MWCO = 3,500) for 12 h with intermittently changing the dialysis fluid and the dialysate was collected in the dialysis bag after filtering through a 0.45-μm pinhole filter membrane. The particle size distribution and zeta potential of the BPseP micelles were determined by Malvern Zetasizer NanoZS, whose morphology images were captured by transmission electron microscope (TEM, Tecnai G2 F20 S-TWIN, 200KV). Absorption spectrum was measured on a UV–Vis 1700 spectrophotometer. berberine-loaded PP micelles (BPP) and blank micelles were prepared with the same procedures. To determine the productivity of BPseP micelles (PB), two factors were calculated. The amount of berberine incorporated in total micelles was calculated by Eq. 1:

1 $${\text{PB}} = \frac{{{\text{weight}}\;{\text{of}}\;{\text{berberine}}\;{\text{in}}\;{\text{micelles}}}}{{{\text{weight}}\;{\text{of}}\;{\text{feeding}}\;{\text{berberine}} + {\text{weight}}\;{\text{of}}\;{\text{feeding}}\;{\text{materials}}}} \times 100\%$$

The efficiency of berberine (EB) utilized in micelles encapsulation was calculated by Eq. 2:

2 $${\text{EB}} = \frac{{{\text{weight}}\;{\text{of}}\;{\text{berberine}}\;{\text{in}}\;{\text{micelles}}}}{{{\text{weight}}\;{\text{of}}\;{\text{feeding}}\;{\text{berberine}}}} \times 100\%$$

### In Vitro Drug Release

The berberine release curve from BPseP micelles was conducted by dialysis method. Generally, each sealed dialysis bag (3500 Da molecular weight cutoff) containing 1 mL BPseP micelles was immersed into 40 mL phosphate buffer saline solution with 10 mM hydrogen peroxide (H_2_O_2_) and phosphate buffer saline solution only (pH 7.4) separately. Subsequently, they were all put into a 37 °C thermostat and their dialysates were collected and supplemented with the same volume of fresh dialysis medium at 0.25, 0.5, 1, 2, 4, 8, 12, 20, 24, 36, 48, and 72 h. All the collected dialysates were measured by an UV–Vis 1700 spectrophotometer to determine the cumulative released berberine from the BPseP micelles, and the average values are presented.

### MTT Assay

In 96-well microplate, 5,000 cells/well were seeded and cultured overnight for cell adhesion and a series concentration of BPseP was administrated to cells. After being incubated for certain time, MTT (5 mg mL^−1^, 10 μL) solution was added to every well. Then, each well was added with 100 μL of MTT dissolving solution (10% SDS and 0.1 mM HCl). Absorbance was measured at 570 (absorbance) and 650 nm (reference). The cell viability was calculated as the percentage of the absorbance of drug-treated wells divided by the absorbance of the control wells.

### ROS and Mitochondrial Superoxide Detection

1 × 10^5^ cells/sample were stained with DCFDA for 15 min at 37 °C in a 5% CO_2_ incubator. After filtration, samples were loaded onto flow cytometer for detection and quantification.

### Immunoblotting

1 × RIPA lysis buffer was supplemented with protease inhibitors and phosphatase inhibitors to form the protein lysis buffer. The concentration of the total protein extract was determined with a Bio-Rad DCTM Protein Assay Kit (Bio-Rad). For detection, 50 μg protein lysate of each samples was loaded onto a 10–12% SDS-PAGE gel and nitrocellulose (NC) membrane was used for protein transfer. After transferring, NC membranes were blocked with 5% milk without fat in TBST for 1 h at room temperature and incubated with the primary antibodies and the secondary antibodies for detection and quantification. GAPDH was used as the loading control.

### RNA Extraction, cDNA Synthesis, and Quantification PCR

RNA was extracted by TRIzol™ reagent (Invitrogen) according to the manufacturer’s instructions. TRIzol™ solution (1 mL) was added to 5 × 10^6^ cells, and the lysate was pipetted up and down several times for homogenization; 0.2 mL of chloroform was added to 1 mL of TRIzol^™^ reagent and centrifuged for 15 min at 12,000 g at 4 °C for protein precipitation. The aqueous phase containing the RNA was transferred to a new tube. Equal volumes of isopropanol were added to the aqueous phase, mixed thoroughly and incubated at − 80 °C for 20 min, and then centrifuged for 30 min at 12,000 g at 4 °C for RNA precipitation. The RNA pellet was re-suspended in 1 mL of 75% ethanol and centrifuged for 5 min at 7,500 × g at 4 °C. The supernatant was discarded and the pellet was air-dried for 5–10 min. RNA pellet was dissolved in 50 μL of RNase-free water. The RNA concentration was determined by using Nano2000 (Thermo Scientific Fisher).

The synthesis of first-strand cDNA was carried out following the instructions of the cDNA synthesis kit (Roche). Briefly, the reaction mixture containing 1 μg RNA, primers, reaction buffer, RNase inhibitor and reverse transcriptase was incubated at 25 °C for 10 min and 55 °C for 30 min. The synthesized cDNA was used for quantification PCR by FastStart Universal SYBR Green Master.

### Immunofluorescence

A sterile cover slide was placed in a 6-well plate. Cells were seeded into the plate and cultured overnight for cell adhesion. After treatment, the cells were fixed with 1 mL of 4% PFA for 15 min and then washed with PBS three times. Triton X-100 (1 mL of 0.1%) was added to the cells and incubated for 5 min to penetrate the cell membrane; the cells were washed three times with PBS. The cells were incubated in Mito Tracker Red staining for 1 h at room temperature in the dark. Finally, cover slides were fixed with Prolong^®^ Gold Anti-fade Reagent with DAPI (Invitrogen). The immunofluorescence images were captured with Confocal Imaging System (Leica Microsystems).

### LC–MS Detection of Berberine

The detection protocol of berberine was reported as previously [21]. Agilent 1290 Infinity UHPLC system was equipped with a binary solvent delivery system and a standard autosampler. The chromatography was performed on a Waters ACQUITY UPLC® BEH C18 column (2.1 × 100 mm, 1.7 µm) (Waters). Mass spectrometry was performed on an Agilent 6230 time-of-flight mass spectrometer (TOF/MS). The results were analyzed by Agilent Bioconfirm protein deconvolution software.

### Oxygen Consumption Rate (OCR)

Firstly, the optimal cell density is required and the density ranges from 3000 to 5000 cells per well. Cells were seeded in Seahorse plate and incubated overnight for cell adhesion at 37 °C. From well B-G, 3000 cells/well was added with 80 µL medium, while wells A and H were only added 80 µL culture medium as control. After being treated for certain time, the cultured medium was replaced by equal volume of assay medium. Oligomycin 10 µM, FCCP 0.5 µM and antimycin A and rotenone 0.5 µM were added to the kit pack to block the complex of mitochondria respiration chain. The OCR was quantified by an Extracellular Flux Analyzer (Seahorse).

### Cell Cycle Analysis

The harvest cells were washed with PBS and fixed with 70% ethanol for 30 min at 4 °C. After fixation, cells were washed twice, spun at 500 g × 5 min and treated with 50 µL of 100 µg mL^−1^ of RNase for each sample. Finally, we added 200 µL PI (50 µg mL^−1^) and stained for 30 min before testing by flow cytometer.

### Adjuvant-Induced Arthritis (AIA) Rat Model

Animal studies were approved by the Ethical Committee of Macau University of Science and Technology. Adjuvant-induced arthritis of rat was induced by complete Freund’s adjuvant (CFA). Briefly, 6–12-week-old Sprague–Dawley (SD) rats were selected and injected with 0.2 mg of heat-killed M. tuberculosis in 100 μL mineral oils through the base of the tail. The rats were divided into 5 groups (7 rats for each group), including healthy control group (without CFA induction), Model group (equal volume of PBS), positive control group (treated with 7.6 mg kg^−1^ MTX/week by oral gavage), berberine 10 mg kg^−1^ and BPseP 1 mg kg^−1^ group administered by intraperitoneal injection. BPseP and berberine were daily administered for a period of 30 days. The severity of arthritis was evaluated by macroscopic inspection. After inflammation is induced, paws are scored on a scale of 0–4, where 0 = normal, 1 = the mildest arthritis, and 4 = the most severe arthritis. The maximum arthritis score is 20 (scoring all four paws and tail).

### Statistical Analysis

All data were calculated as the mean ± SEM for triplicate individual experiments. Differences between groups were determined using a one-way analysis of variance (ANOVA) using GraphPad Prism 7. Student’s t test was used to compare two groups. The level of significance was set at *P* < 0.05 for all tests.

## Results and Discussion

### ROS for Developing Specific Drug Delivery in RA Fibroblast Cells

**Fig. 1:**
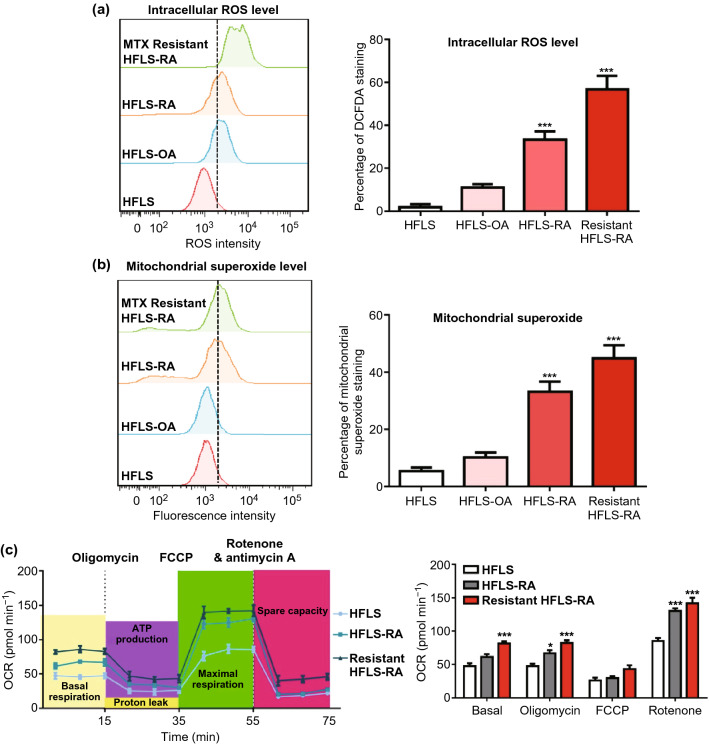
ROS level is associated with the progress of RA. **a**, **b** ROS and mitochondrial superoxide levell were detected and compared in MTX-resistant, RA, OA, and normal synovial fibroblast. **c** OCR was evaluated in MTX-resistant HFLS-RA, HFLS, and HFLS-RA. Data were analyzed as the mean ± SEM for triplicate individual experiments (*P < 0.05, **P < 0.01, ***P < 0.001)

To facilitate the preferential accumulation of berberine in RA-affected cells, we intended to develop targeted nanotherapy. Since high ROS is a representative characteristic of inflammatory microenvironment, to explore whether it could be used for specific drug delivery, we compared the ROS level of different types of primary synoviocytes: human fibroblast-like synoviocytes (HFLS), HFLS-osteoarthritis (HFLS-OA), HFLS-rheumatoid arthritis (HFLS-RA) and MTX-resistant HFLS-RA. The highest ROS level was observed in MTX-resistant fibroblast, the RA fibroblast took the second place and the normal synovial fibroblast was the lowest (Fig. 1a). It indicates that ROS level is closely associated with the progress of RA.

Next, because the anti-RA mechanism of berberine was through targeting mitochondria, we investigated whether ROS-responsive delivery is suitable for targeting mitochondria and the level of superoxide and oxygen consumption rate (OCR) were evaluated in the above cells. As a result, the trend of these two indicators in different cells is similar to ROS (Fig. 1b, c): MTX-resistant cells are followed by RA cells, and the lowest is healthy fibroblast. Moreover, the expression level of SOD1 and SOD2, which is the major scavenger of mitochondrial ROS [23], is significantly increased in RA fibroblast cells (Fig. S1a). Therefore, theoretically ROS-responsive delivery system will be qualified for mitochondrial targeting delivery. At last, we detected the oxidative level of clinical serum samples (the ratio of GSSG/GSH). Consistent with cellular results, the highest oxidative stress was acquired in MTX-resistant patient samples and higher level of ROS was observed in RA patients than healthy donors (Fig. S1b).

Taken together, based on the difference of cellular ROS and mitochondrial superoxide among HFLS, HFLS-RA and MTX-resistant HFLS-RA, ROS-responsive drug delivery system could be a potential method to enhance the efficacy in RA.

### Drugs Preparation and Release In Vitro

**Fig. 2:**
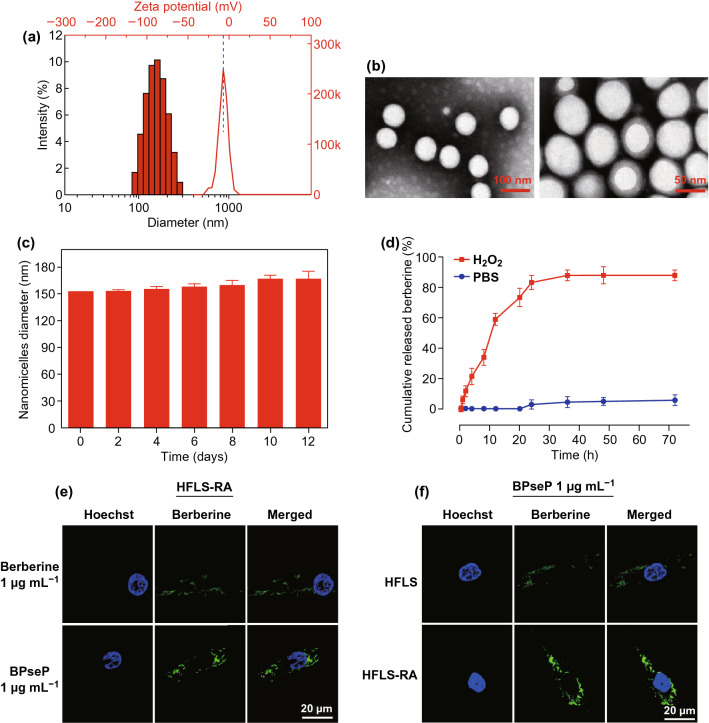
**a** Average particle size and zeta potential of BPseP. **b** A uniform spherical morphology of nanoparticles. **c** BPseP could maintain stability at 37 °C for 2 weeks. **d** BPseP micelles could cumulatively release berberine quickly more than 80% because the diselenium bond in the PseP structure is sensitive to the ROS. **e** BPseP significantly up-regulated the cellular accumulation of berberine. **f** Compared with HFLS, HFLS-RA induced much berberine intracellular accumulation. Data were analyzed as the mean ± SEM for triplicate individual experiments (*P < 0.05, **P < 0.01, ***P < 0.001)

The ROS-responsive berberine polymeric micelles (BPseP) were designed as shown in Figs. S1c, d and S2a, b. Our schematic illustration displayed that BPseP passively diffused to tissues, but in inflammation areas, high concentration of oxidative substrates could irritate the cleavage of the mPEG–Se–Se–PLGA amphiphilic co-polymers and berberine was released to produce higher ROS, which further facilitates the collapse of micelles. After preparation of the BPseP micelles, we used dynamic light scattering (DLS) methods to characterize its hydrodynamic diameter. Its average particle size is about 153 nm, with polydispersity index (PDI) of 0.059, zeta potential of − 5.12 mV (Fig. 2a) and a uniform spherical morphology (Fig. 2b). The micelles could maintain stability at 37 °C for 2 weeks (Fig. 2c). Its drug loading capacity is 28.75%, and the berberine encapsulation efficiency of the as-prepared BPseP micelles is 86.25%. From Fig. 2d, we could obviously distinguish the different responses of BPseP micelles upon different oxidation circumstances such as 10 mM H_2_O_2_ mimicking oxidative situation (high ROS) in rheumatoid arthritis tissues and PBS only like normal cell condition, in which BPseP micelles could cumulatively release berberine quickly more than 80% because the diselenium bond in the PseP structure is sensitive to the ROS.

### Cellular Uptake and In Vitro Anti-RA Activity of Berberine

**Fig. 3:**
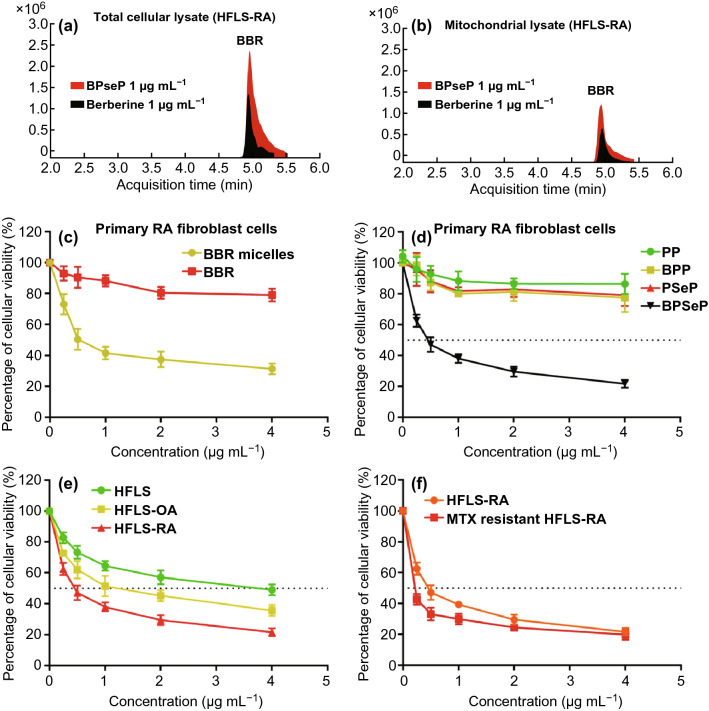
**a**,** b** LC–MS results showed that cellular and mitochondrial concentration of berberine was greatly increased by BPseP, when compared with berberine administration. **c** The efficacy of BPseP and berberine on primary HFLS cells. **d** BPseP effectively inhibited the growth of cells, and its IC_50_ value is around 0.6 ug mL^−1^. For other parallel controls, no significant inhibitory effect was observed. **e** The efficacy of BPseP among different types of HFLS was detected. **f** Compared with HFLS-RA, IC_50_ of BPseP in MTX-resistant HFLS is much lower. Data were analyzed as the mean ± SEM for triplicate individual experiments (*P < 0.05, **P < 0.01, ***P < 0.001)

To investigate whether the BPseP micelles could promote the cellular uptake of berberine in RA, firstly we utilized the autofluorescence of berberine to determine its accumulation. As shown in Figs. 2e, f and S3, BPseP remarkably increased the cellular uptake of berberine, and compared with HFLS, the fluorescence intensity is much higher in HFLS-RA which contains high ROS level. Meanwhile, in Fig. 3a, b, LC–MS results also presented that both cellular and mitochondrial concentrations of berberine were greatly increased by BPseP, when compared with berberine administration. The result is consistent with their discriminated efficacy on primary HFLS cells (Fig. 3c). Next, the cytotoxic effect of BPseP was determined on various types of HFLS. ROS-responsive nano-micelles without berberine (PseP), nonresponsive nano-micelles with or without berberine (PP and BPP) were applied as control. As shown in Fig. 3d, BPseP effectively inhibited the growth of cells and its IC_50_ value is around 0.6 μg mL^−1^. For other parallel controls, no significant inhibitory effect was observed. The efficacy of BPseP among different types of HFLS was detected as well. HFLS-RA is the most sensitive one and the following is HFLS-OA, while HFLS shows the lowest cytotoxic effect (Fig. 3e). Furthermore, compared with HFLS-RA, IC_50_ of BPseP in MTX-resistant HFLS is lower (Fig. 3f). Therefore, BPseP could effectively promote the cellular accumulation and in vitro anti-RA activity of berberine and thus enhanced the efficacy.

### Anti-RA Effect of BPseP Micelles

**Fig. 4:**
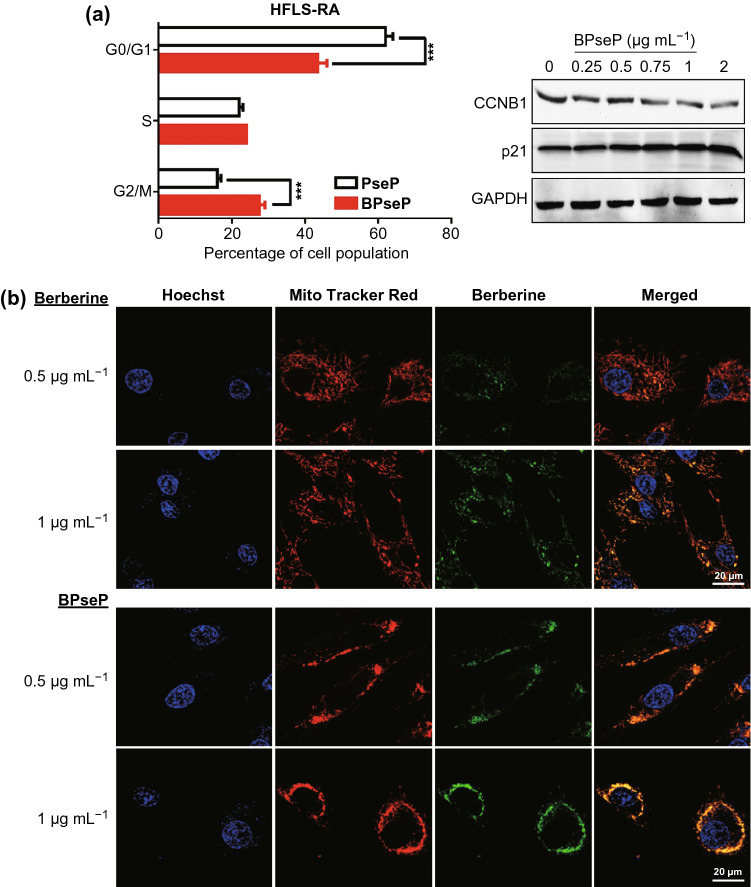
**a** BPseP significantly caused G2 arrest, down-regulation of CCNB1 and the increase of p21. **b** Immunofluorescence detection of berberine and mitochondria. The intracellular location of berberine was mostly overlapped with mitochondria. Data were analyzed as the mean ± SEM for triplicate individual experiments (*P < 0.05, **P < 0.01, ***P < 0.001)

**Fig. 5:**
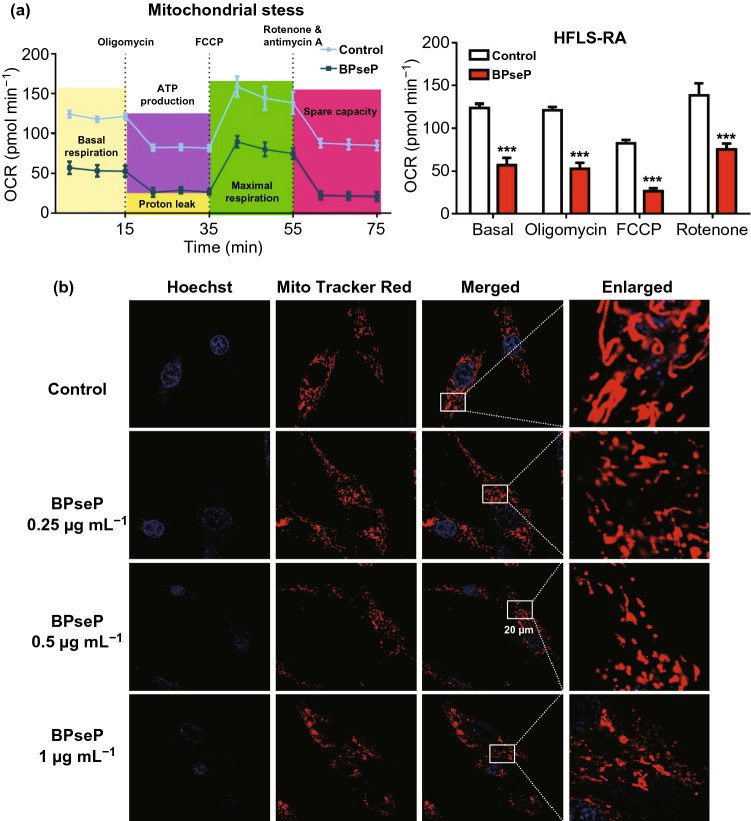
**a** OCR was remarkably suppressed by BPsep in HFLS-RA. **b** With the increasing concentration of BPseP, the morphology change of mitochondria became shorter and smaller. Data were analyzed as the mean ± SEM for triplicate individual experiments (*P < 0.05, **P < 0.01, ***P < 0.001)

As we have reported previously that berberine suppressed RA through directly interacting with respiration chain complex I and inducing cell cycle arrest [21], we investigated the effect of BPseP on cell cycle and mitochondrial function. In Fig. 4a, BPseP significantly caused G2 arrest and down-regulation of G2 regulator CCNB1. Next, we utilized the autofluorescence of berberine to determine its intracellular location. Mito Tracker Red staining was used to visualize mitochondria. As shown in Fig. 4b, the fluorescence of berberine was mostly overlapped with mitochondria. Oxygen consumption rate as an important indicator of mitochondrial function was remarkably suppressed by BPsep in HFLS-RA (Fig. 5a). The morphology change of mitochondria was observed as well. With the increasing concentration of BPseP, the mitochondria became shorter and smaller (Fig. 5b). Mitochondrial superoxide which is the by-product of mitochondrial respiration was enhanced by BPseP, while the levels of two mitochondrial ROS scavengers SOD1 and SOD2 were inhibited (Fig. S4a). These results demonstrated that BPseP could effectively gather berberine in mitochondria and thus enhance the in vitro efficacy.

### BPseP-Induced anti-RA Effect

**Fig. 6:**
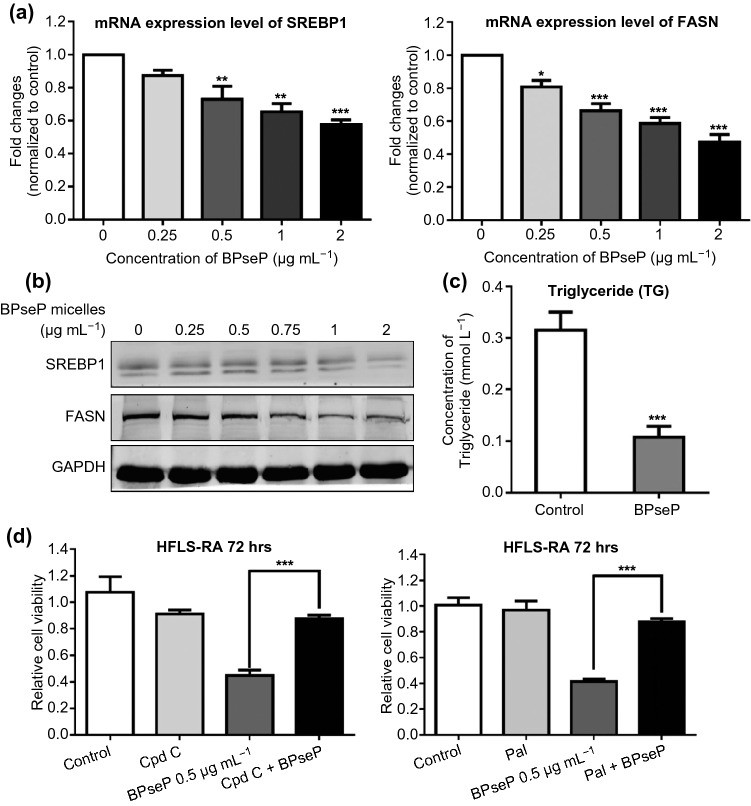
**a**, **b** SREBP1 and FASN were significantly down-regulated by the treatment of BPseP. **c** The cellular level of TG was decreased. **d** AMPK inhibitor compound C and palmitic acid (the key intermediator of lipogenesis) partially counteract the suppressive effect of BPseP. Data were analyzed as the mean ± SEM for triplicate individual experiments (*P < 0.05, **P < 0.01, ***P < 0.001)

As matter of fact, mitochondria is the energy generator and if mitochondrial function was inhibited, the energy sensor AMPK will be activated and suppress downstream anabolism [24, 25]. As we have been proved that berberine inhibited the cell growth through activating AMPK and suppressing lipogenesis, the effect of BPseP on AMPK and lipogenesis was detected. As shown in Figs. S4b and S5a, AMPK signaling pathway was remarkably activated and two key transcription factors of lipogenesis SREBP1 and FASN were significantly down-regulated (Fig. 6a, b). Moreover, the cellular level of triglyceride (TG) which is a representative component of lipid was decreased (Fig. 6c). At last, we used AMPK inhibitor compound C and palmitic acid (the key intermediator of lipogenesis) to counteract the suppressive effect of BPseP. Both of them greatly rescued cells from death (Fig. 6d). Therefore, we concluded that the anti-RA effect of BPseP was achieved by activating AMPK and inhibiting lipogenesis.

### Enhancement of Efficacy of Berberine In Vivo

**Fig. 7:**
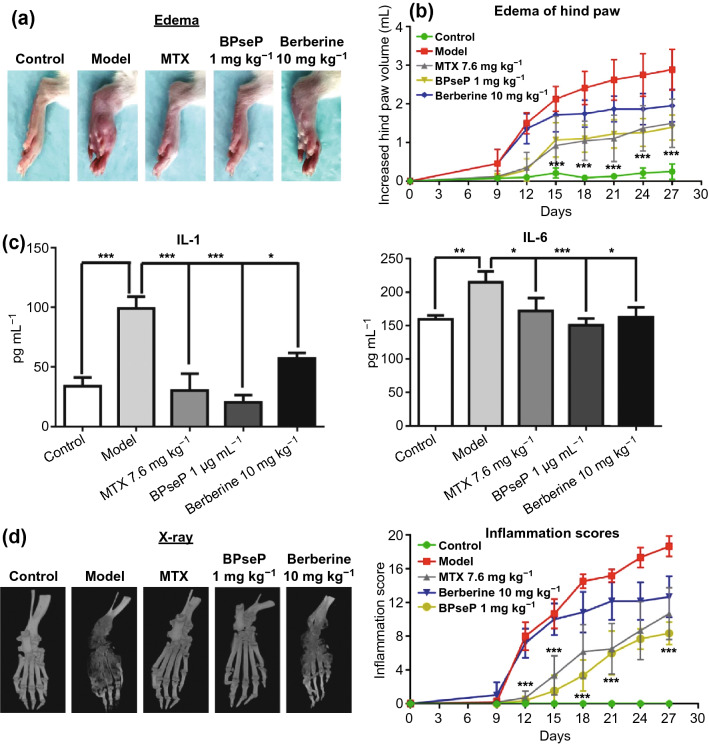
**a**,** b** BPseP treatment acquired a significant regression in edema of the hind paw. **c** BPseP remarkably inhibited the secretions of IL-1 and IL-6. **d** The results of X-ray imaging showed that BPseP is able to protect the joint bone from destruction induced by AIA. Data were analyzed as the mean ± SEM for triplicate individual experiments (*P < 0.05, **P < 0.01, ***P < 0.001)

The in vivo efficacy of BPseP was investigated in adjuvant-induced arthritis (AIA) model of Sprague–Dawley rats. The experiment was divided into five groups: healthy, AIA model and three treatment groups MTX 7.6 mg kg^−1^ (positive control)/BPSeP micelles 1 mg kg^−1^/berberine 10 mg kg^−1^ (parallel control). The severity of arthritis was evaluated by three indicators: edema of hind paw, increase of serum cytokines, and bone destruction. As shown in Fig. 7a, b, BPseP achieved similar effect with MTX in attenuating the edema of hind paws of AIA rat. Both of treatments acquired a significant regression in edema of the hind paw. Attractively, the promising therapeutic efficacy of BPseP was also observed in inhibiting cytokines generation and bone damage. It remarkably inhibited the secretions of IL-1 and IL-6, which are the key cytokines related to inflammation (Fig. 7c), and the results of X-ray imaging demonstrate that BPseP is able to protect the joint bone from destruction induced by AIA (Fig. 7d). To conclude, BPseP is an effective agent in preventing inflammation and even bone damage caused by arthritis. However, with berberine as the parallel control, the treatment with 10 mg kg^−1^ dosage was able to suppress the release of inflammatory cytokines, but it failed to suppress the edema and bone destruction. These results indicated that BPseP achieved much better efficacy than berberine, even at one-tenth concentration.

Furthermore, the effect of BPseP on regulation activity of immune cells was analyzed. Since hyper-activation of CD8^+^ effector T cells and suppression of Treg are two main reasons that are responsible for the development of autoimmune symptom and tissue damage [26–28], the changes in these two kinds of cells were investigated. As shown in Fig. S5b, BPseP could significantly up-regulate the percentage of Treg and meanwhile lower the activity of CD8^+^ cells.

Taken together, BPseP is a potential agent for anti-RA in vivo study, no matter in protecting tissue from damage or down-regulating the response of immune cells.

## Conclusions

As an autoimmune disease, the innate immune system is persistently activated in RA [29] and continuously expressed cytokines such as IL-1 and IL-6 [30]. As has been validated i*n vivo* study, BPseP significantly suppressed the inflammation, cytokines production, and even the bone destruction and most intriguingly, BPsep showed much better efficacy than berberine, which suggested that ROS-responsive delivery is conducive to anti-RA therapy. Moreover, BPseP showed powerful effect on restoring the balance between suppressive and effective immune cells. Regulatory T cells (Treg), as the major subpopulation of suppressor T cells, which is thought to play an important role in attenuating RA and preventing autoimmune disease [31], were substantially up-regulated by BPseP, whereas the activity of CD8^+^ effector T cells was down-regulated. Notably, besides the suppressive effect on HFLS-RA, BPseP is able to modulate and restore the immune balance in RA. In sum, the ROS-responsive micelles could preferentially bond to mitochondria of inflammatory tissue to kill affected cells and showed tenfold higher efficacy than that of the berberine. Therefore, those rationally designed ROS-responsive nanoparticles of berberine provided a feasible approach to improve drug accumulation in RA-injured tissues and achieve satisfied therapeutic efficacy. It is worth noting that, in the future, the efficiency of targeting therapy of responsive micelles could be further enhanced by integrating with targeting antibody, such as anti-TNF, which can promote binding with RA-affected cells.

## Electronic supplementary material

Below is the link to the electronic supplementary material.

Supplementary file1 (PDF 786 kb)
