# Supplementary material for: ROS-Responsive Berberine Polymeric Micelles Effectively Suppressed the Inflammation of Rheumatoid Arthritis by Targeting Mitochondria
Source: Nanomicro Lett. 2020 Mar 20;12:76. doi: 10.1007/s40820-020-0410-x (PMC7770724; doi:10.1007/s40820-020-0410-x)
Supplement: Supplementary file 1 — Supplementary file1 (PDF 786 kb) [file 40820_2020_410_MOESM1_ESM.pdf]

Supporting Information for

ROS-responsive Berberine Polymeric Micelles Effectively Suppressed the Inflammation of Rheumatoid Arthritis by Targeting Mitochondria

Xing-xing Fan[1, a], Meng-ze Xu[2, a], Elaine Lai-Han Leung[1], Cai Jun[1], Zhen Yuan[2, *], Liang Liu[1, *]

[1]State Key Laboratory of Quality Research in Chinese Medicine, Macau Institute For Applied Research in Medicine and Health, Macau University of Science and Technology, Avenida Wai Long, Taipa, Macau, SAR, China

[2]Faculty of Health Science, University of Macau, Macau, SAR, China

[a]Xing-xing Fan and Meng-ze Xu contributed equally to this work

*Corresponding authors. E-mail: lliu@must.edu.mo (Liang Liu); zhenyuan@um.edu.mo (Zhen Yuan)

Supplementary Figures

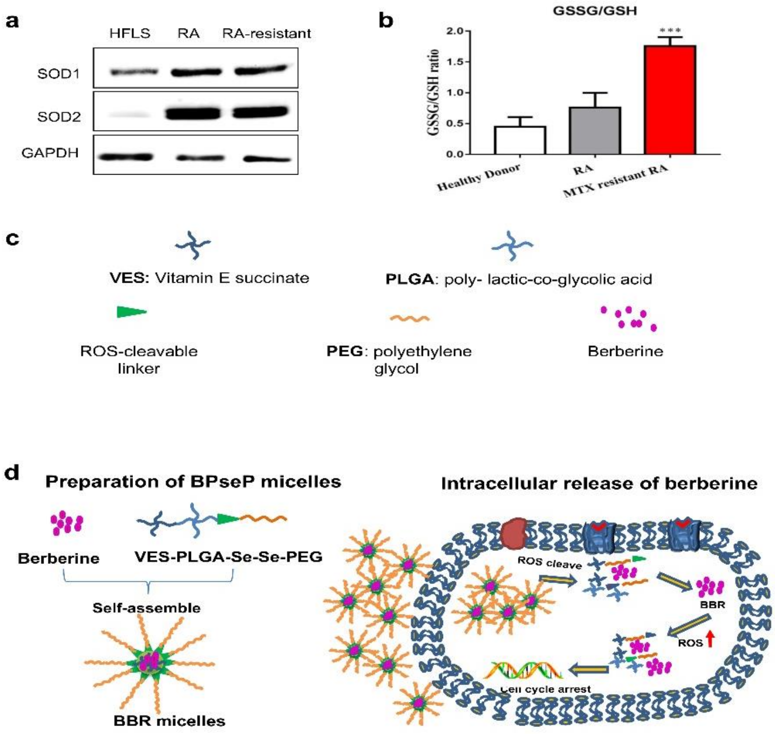

Fig. S1 (a) The expression level of SOD1 and SOD2 was significantly increased in RA fibroblast cells. **(b)** The ratio of GSSG/GSH in serum samples was detected in MTX-resistant patient samples, RA patients and healthy donors. **(c)** The components of BPseP. **(d)** Schematic illustration of BPseP self-assembling and ROS-responsive

release mechanism. Data were analysed as the mean ± SEM for triplicate individual experiments (*$^*P < 0.05$, $^{**}P < 0.01$, $^{***}P < 0.001$*).

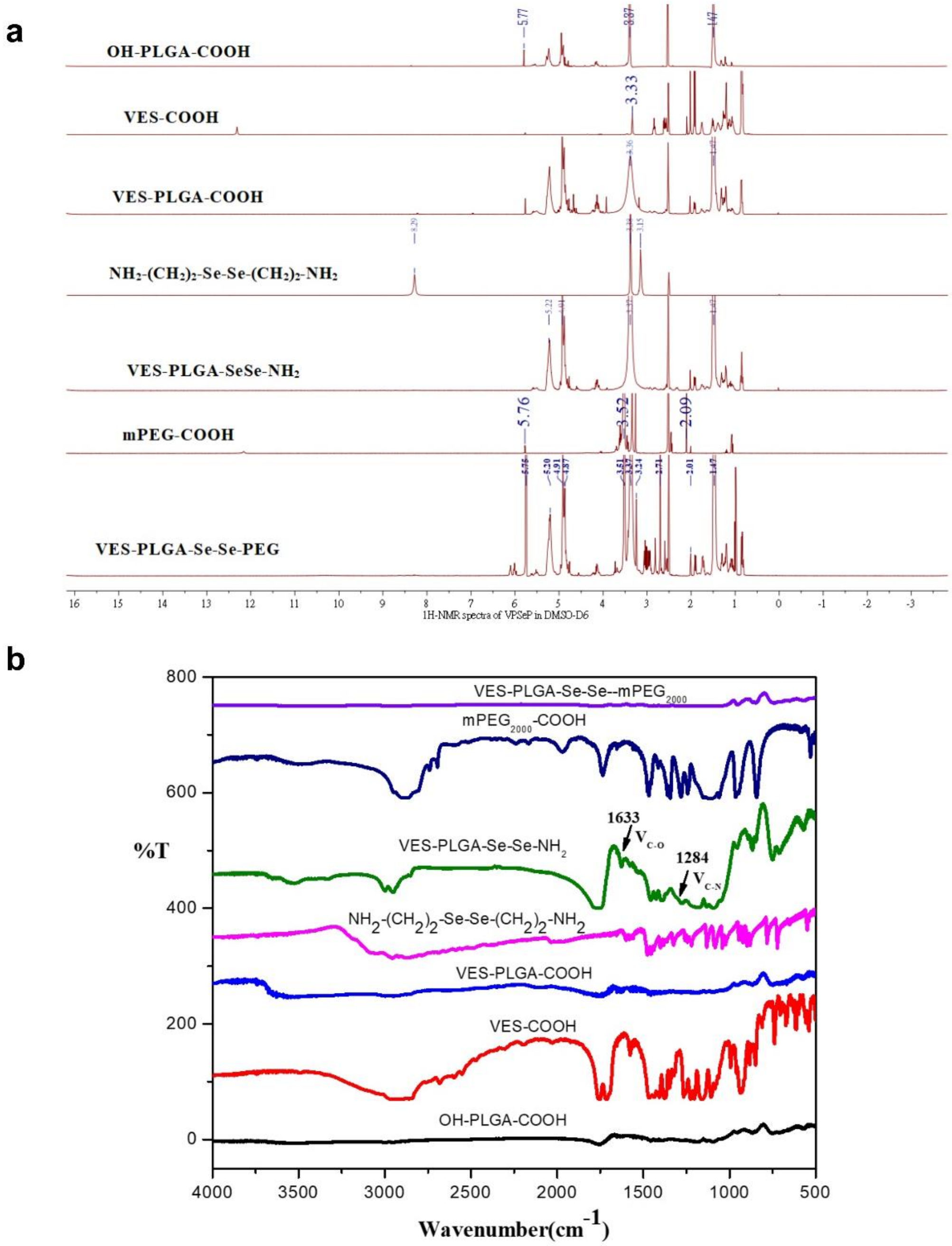

Fig. S2 (a) The structures of VES-PLGA-Se-Se-PEG, mPEG-COOH, VES-PLGA-Se-Se-NH2, NH2-(CH2)2-Se-Se-(CH2)2-NH2, VES-PLGA-COOH, VES-COOH, OH-PLGA-COOH were all characterized by 1H nuclear magnetic resonance (1H-NMR), in which the characteristic peak at around δ 3.51 ppm, 5.20 ppm, 3.37 ppm and the peaks below 2.01 ppm was corresponding to the PEG, selenocystamine dihydrochloride (NH2-(CH2)2-Se-Se-(CH2)2-NH2), Vitamin E succinate and PLGA block, respectively. **(b)** The FT-IR spectra of VES-PLGA-Se-Se-NH2 revealed that

the stretching bands at 1633 cm-1 and 1284 cm-1 denoted the amide bond in VES-PLGA-Se-Se -NH2, and the FT-IR spVES-PLGA-ectra summarization of VES-PLGA-Se-Se-PEG, mPEG-COOH, Se-Se-NH2, NH2-(CH2)2-Se-Se-(CH2)2-NH2, VES-PLGA-COOH, VES-COOH, OH-PLGA-COOH combined with the 1H-NMR results demonstrated that VES-PLGA-Se-Se-PEG were successfully synthesized.

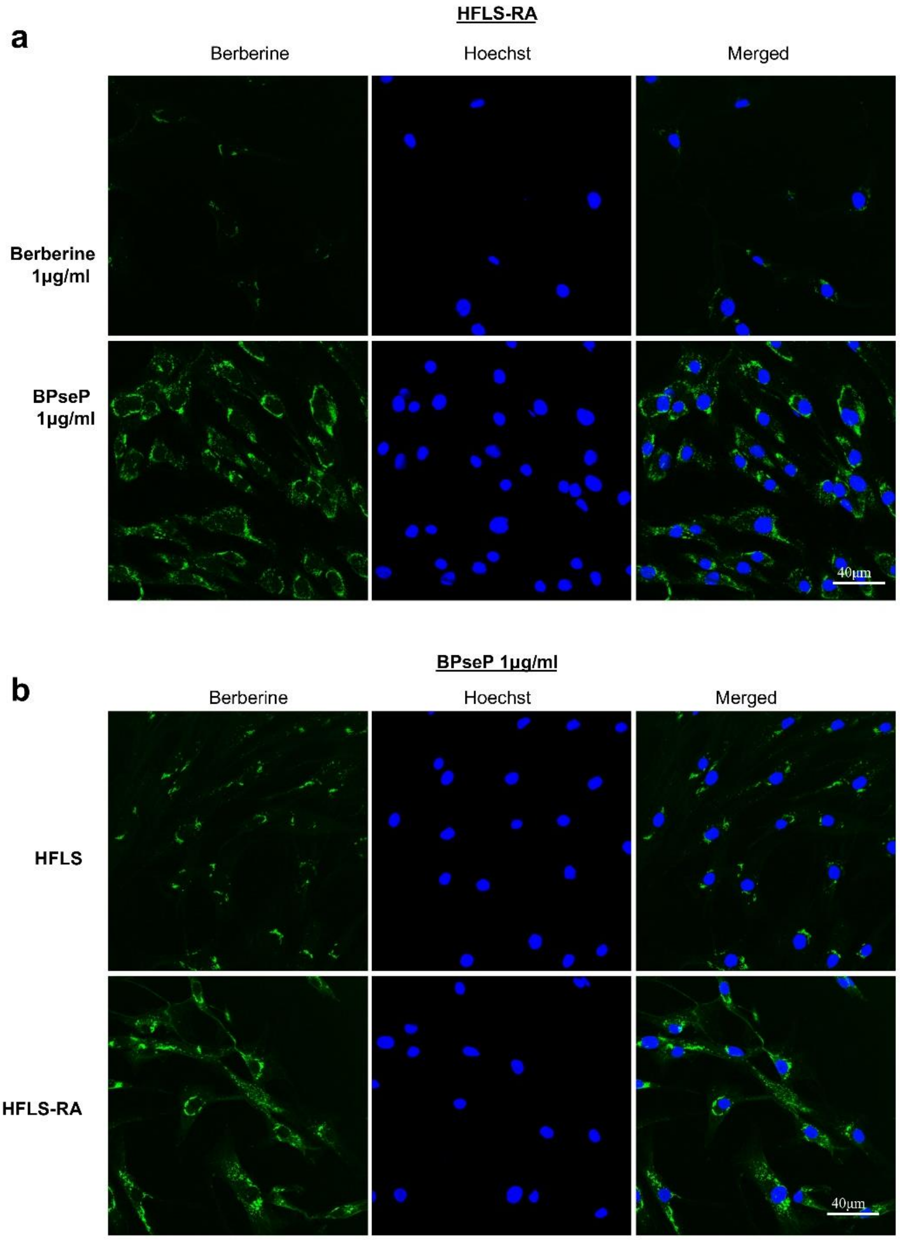

Fig. S3 (a) The Immunofluorescence results showed BPseP significantly up-regulated the cellular accumulation of berberine in HFLS-RA. **(b)** Although cells were treated with the same concentration of BPseP, HFLS-RA induced much berberine accumulated.

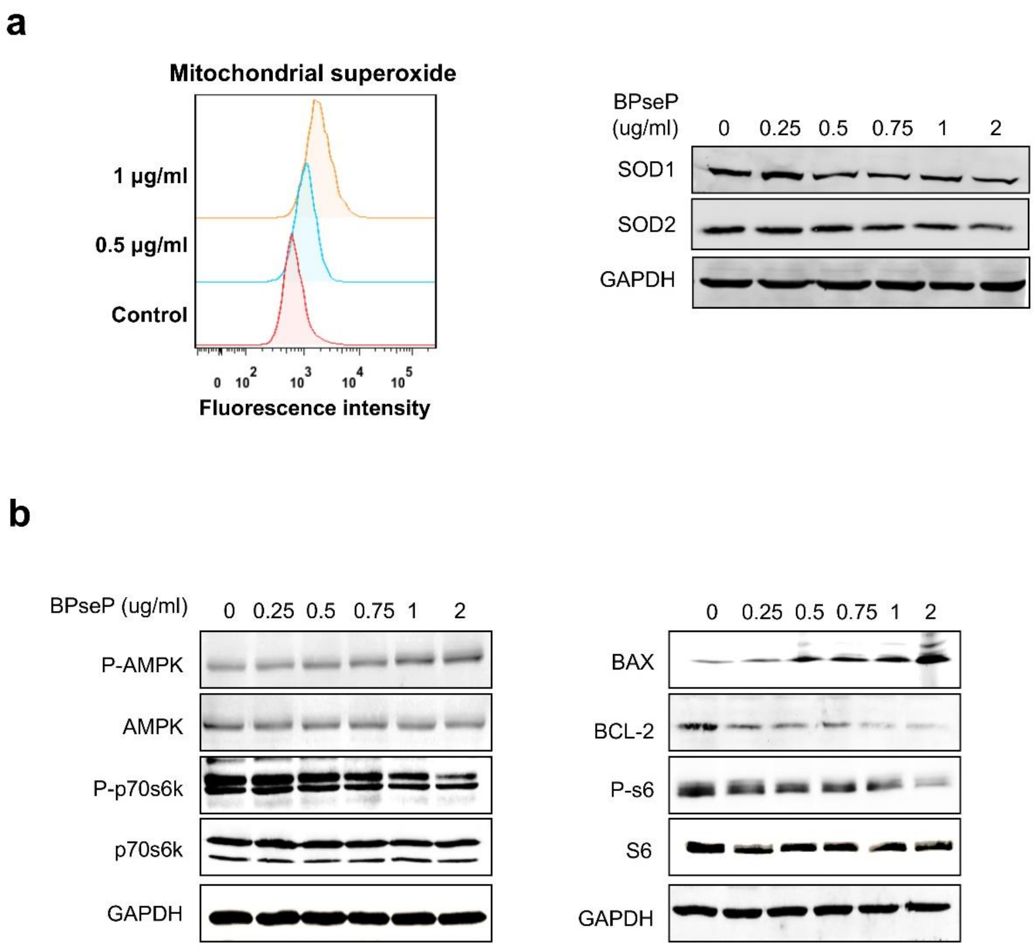

Fig. S4 (a) Mitochondrial superoxide was enhanced by BPseP, while the level SOD1 and SOD2 were inhibited. **(b)** AMPK signaling pathway was activated by BPseP

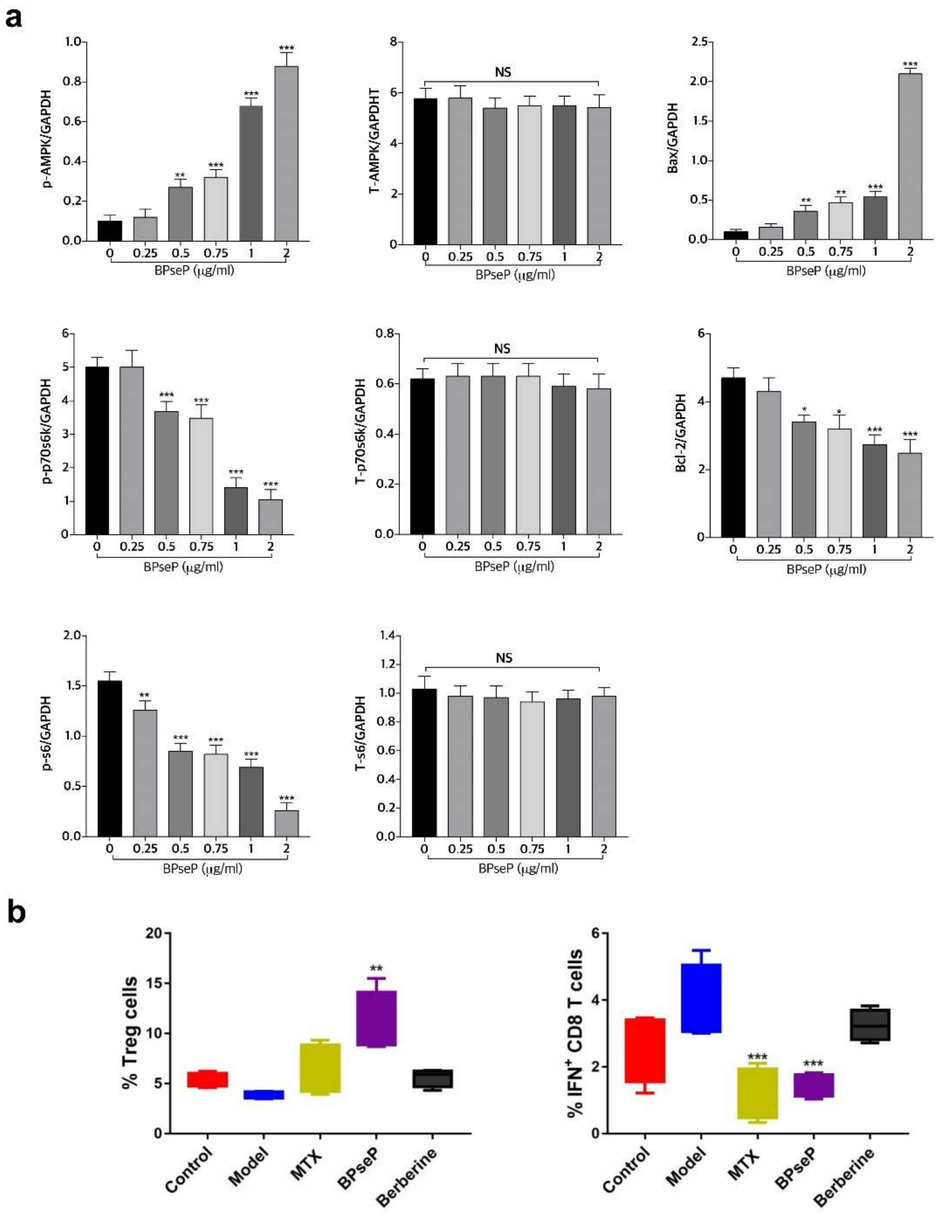

Fig. S5 (a) The quantitative statistical data of repeated experiments of Supplementary Fig 4b. **(b)** BPseP significantly up-regulated the percentage of Treg and meanwhile decreased the activity of $CD8^{+}$ cells
